# Fluorescence Melting Curve Analysis for Concurrent Genotyping of Three Tag SNPs in *FUT3*

**DOI:** 10.3390/diagnostics12123039

**Published:** 2022-12-04

**Authors:** Mikiko Soejima, Yoshiro Koda

**Affiliations:** Department of Forensic Medicine, Kurume University School of Medicine, Kurume 830-0011, Japan

**Keywords:** fluorescence melting curve analysis, *FUT3*, Lewis-negative allele, rs28362459, rs778986, rs28362463

## Abstract

The synthesis of Lewis blood group antigens is governed by two fucosyltransferase genes, *FUT2* and *FUT3*. Evidence is accumulating to suggest that functional polymorphisms of *FUT2* and *FUT3* are associated with a variety of clinical conditions. Fluorescence melting curve analysis (FMCA), using three different dual-labeled probes for concurrent genotyping of three single nucleotide polymorphisms (SNPs) of *FUT3*, c.59T>G, c.314C>T, and c.484G>A for Lewis-negative allele inference, was developed and validated using Ghanaian and Caucasian subjects. Although two other SNPs, c.55G>A, and c.61C>T, are located in the probe sequence for c.59T>G, it seems feasible to detect these two SNPs along with c.59T>G. The results obtained by probe-based FMCA were in perfect accordance with those obtained by Sanger sequencing for 106 Ghanaians and 100 Caucasians. The present method is useful and reliable for estimating Lewis-negative alleles on a relatively large scale.

## 1. Introduction

The Lewis blood group antigens are the ABO(H) blood group-related antigens, consisting of Lewis a (Le^a^) and Lewis b (Le^b^) antigens, which are present not only on red blood cells, but also in other tissues and body fluids, such as saliva [1]. Le antigens on red blood cells are thought to be acquired secondarily from plasma [2]. The synthesis of these antigens is governed by *FUT2*-encoded α(1,2)fucosyltransferase (Se enzyme) and *FUT3*-encoded α(1,3/1,4)fucosyltransferase (Le enzyme). The functional alleles, *Se* and *Le*, are dominant over the nonfunctional alleles, *se* and *le*. Thus, subjects homozygous for *le*, i.e., Le-negative individuals, show a Le(a-b-) phenotype regardless of secretor status. While subjects with at least one *Le* (*Le/Le* or *Le/le*), i.e., Le-positive individuals, show an Le(a−b+) phenotype in secretors (*Se/Se* or *Se/se*), Le(a+b−) in non-secretors (*se/se*), and Le(a+b+) in weak-secretors (*Se^w^*/*Se^w^* or *Se^w^*/*se*) [3]. Conventional serological Le phenotyping is somewhat difficult because it depends on the strength and specificity of the anti-Le^a^ and anti-Le^b^ antibodies used and the skill of the observer [4]. Therefore, phenotypic inference by reliable *FUT2* and *FUT3* genotyping is a useful alternative method for Le phenotyping.

The *FUT3* polymorphism shows a population-specific distribution [5,6], and there are several Le enzyme-inactivating SNPs within a population. As described previously, four SNPs, c.202T>C (p.Trp68Arg, rs812936), c.484G>A (p.Trp68Arg, rs28362463), c.508G>A (p.Gly170Ser, rs3745635), and c.1067T>A (p.Ile356Lys, rs3894326), are the major causal SNPs for Le enzyme inactivation. Each of them is in linkage disequilibrium with other SNPs [5,6,7,8,9,10]: c.202T>C is in near absolute linkage disequilibrium with c.314C>T (p.Thr105Met, rs778986) (the allelic distributions are almost completely dependent on each other, i.e., T-C and C-T combinations are common for 202–314, but T-T and C-C combinations rarely exist), and c.484G>A is also in near absolute linkage disequilibrium with c.13G>A (p.Gly5Ser, rs28362458) [7,8,9]. In addition, c.59T>G (p.Leu20Arg, rs28362459) is closely linked with c.508G>A or c.1067T>A. According to the Erythrogene database (https://www.erythrogene.com/) [11], *FUT3* alleles with 59G and 508A account for 15.2%, and those with 59G and 1067A for 9.4%, while those with 59T and 508A account for 0.14%, and those with 59T and 1067A for 0.22%, in the global population. Furthermore, the rare 59G allele, with neither 508A nor 1067A (0.68% in global population), has been reported to exhibit a Le(a-b-) erythrocyte phenotype but is positive for Lewis antigen in saliva [12]. Therefore, 484G>A (or c.13G>A), c.59T>G (or c.508G>A plus c.1067T>A), and c.202T>C (or c.314C>T) are suggested to be useful for estimating *le* alleles in many populations [5,6,13,14].

Fluorescence melting curve analysis using oligonucleotide probes (probe-based FMCA) is one of the most robust methods to detect SNPs [15]. Dual-labeled probes are commonly used for hydrolysis-probe (TaqMan) assay but can also be used for FMCA and allow multiplex assay by using different fluorochromes [16]. In the present study, we developed and validated probe-based FMCA for concurrent genotyping of c.59T>G, c.314C>T, and c.484G>A of *FUT3*.

## 2. Materials and Methods

The ethical committee of Kurume University reviewed and approved the research protocol (bioethics approval number 342).

### 2.1. DNA Samples

Genomic DNA previously isolated from 106 Ghanaians in Accra and genomic DNA from 100 Caucasians (HD100CAU) purchased from the Coriell Institute (Camden, NJ) were used for this study. The *FUT3* coding sequences of these samples had already been determined by Sanger sequencing [13].

### 2.2. Probe-Based FMCA for Concurrent Genotyping of c.59T>G, c.314C>T, c.484G>A of FUT3

**Figure 1:**
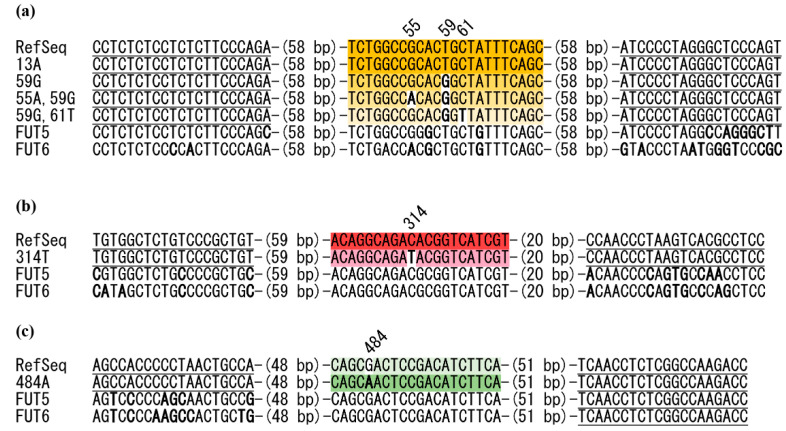
Alignment of DNA sequences of three amplicons and dual-labeled probes of the triplex probe-based FMCA for detection of c.59T>G, c.314C>T, and c.484G>A of *FUT3*. Reference sequence (RefSeq), derived alleles of *FUT3*, and corresponding sequences of *FUT5* and *FUT6* are aligned. Positions of the SNPs are shown in the upper sequences. Primer sequences are underlined, and dual-labeled probes are shaded green for FAM, yellow for HEX, and red for Cy5. Bold letters indicate nucleotides that differ from the reference sequence. (**a**): Alignment of DNA sequences around c.59T>G. In addition to three target SNPs, c.55G>A and c.61C>T are also shown because these sites are polymorphic in the 59T-probe. (**b**): Alignment of DNA sequences around c.314C>T. (**c**): Alignment of DNA sequences around c.484G>A.

Since the human genome contains three paralogous genes with high sequence similarity, *FUT3*, *FUT5*, and *FUT6* [17,18], primers should be designed to amplify only *FUT3*, not *FUT5* and *FUT6*. Three pairs of primers for amplification of three regions containing each of c.59T>G, c.314C>T, and c.484G>A, respectively, and three self-quenching probes labeled with different fluorophores (59T-, 314C-, and 484A-probes) to determine c.59T>G, c.314C>T, and c.484G>A, are indicated in Figure 1 and Table 1.

To amplify the region containing c.484G>A, the same PCR primers as the Eprobe-based FMCA for detection of c.508G>A were used [19]. The asymmetric PCR reaction mixture has a final volume of 10 µL and contains 5 µL of Premix Ex Taq (Probe qPCR) (Takara, Tokyo, Japan), 50 nM of 59F-primer, 500 nM of 59R-primer, 50 nM of 314F-primer, 500 nM of 314R-primer, 50 nM of 484F-primer, 500 nM of 484R-primer, 100 nM of 59T-probe, 200 nM of 314C-probe, 200 nM of 484A-probe, and 2–20 ng of genomic DNA. The PCR was conducted on a LightCycler 480 instrument II (Roche Diagnostics, Tokyo, Japan) with the following thermal conditions: pre-denaturation at 95 °C for 30 s, then 50 cycles of denaturation at 95 °C for 5 s, and annealing/extension at 60 °C for 15 s. The PCR products were then heated to 95 °C for 1 min, cooled to 40 °C for 1 min, and fluorescence data were obtained during heating from 40 to 80 °C at a 0.1 °C/s ramp rate with 3 acquisitions/s using the FAM filter (Excitation-Emission: 465 nm–510 nm) for the 484A-probe, VIC/HEX/Yellow 555 filter (533 nm–580 nm) for the 59T-probe and Cy5/Cy5.5 filter (618 nm–660 nm) for the 314C-probe. Melting curve genotyping and melting temperature (Tm) calling analyses were carried out using LightCycler 480 gene scanning software.

## 3. Results

### 3.1. Probe-Based FMCA for Genotyping of c.59T>G, c.314C>T, and c.484G>A of FUT3

In this study, we first considered five SNPs, c.13G>A, c.59T>G, c.202T>C, c.314C>T, and c.484G>A, of *FUT3*, as candidates for probe-based FMCA analysis to infer le alleles in various populations. Among them, c.484G>A and c.314C>T showed more favorable analysis results than c.13G>A and c.202T>C, respectively, which could be the respective surrogate SNPs. Accordingly, the three SNPs—c.59T>G, c.314C>T, and c.484G>A—were finally selected for le allele inference (data not shown). Therefore, in this study, a triplex probe-based FMCA analysis for c.59T>G using a 179-bp amplicon, HEX-labeled 59T-probe, for c.314C>T using a 140-bp amplicon and Cy5-labeled 314C-probe, and for c.484G>A using a 158-bp amplicon and FAM-labeled 484A-probe, was carried out (Figure 1a–c).

**Figure 2:**
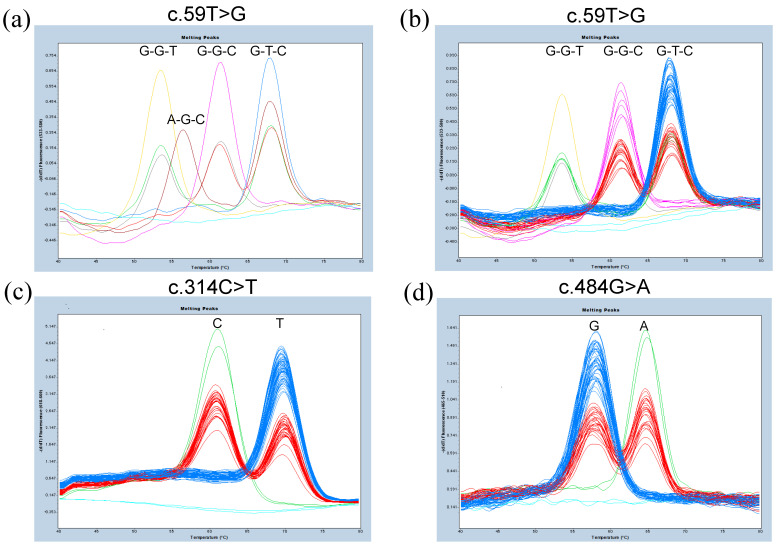
Melting peak profiles of probe-based FMCA for detection of SNPs of *FUT3*. (**a**) Melting peak profiles of probe-based FMCA for detection of 59T>G of seven selected subjects whose genotypes were already determined to be 55G>A, 59T>G, and 61C>T. Subjects with genotypes of G-T-C/G-T-C at 55-59-61 are shown in blue, G-T-C/G-G-C in red, G-G-C/G-G-C in pink, G-T-C/G-G-T in green, G-G-C/G-G-T in gray, G-G-T/G-G-T in yellow, and G-T-C/A-G-C in brown. Negative control is shown in light blue. (**b**) Melting peak profiles of probe-based FMCA for detection of 59T>G of 76 Ghanaian subjects. (**c**) Melting peak profiles of probe-based FMCA for detection of c.314C>T of 90 Caucasians whose genotypes were known. The subjects with genotypes of C/C at c.314C>T are shown in blue, C/T in red, T/T in green. Negative control is shown in light blue. (**d**) Melting peak profiles of probe-based FMCA for detection of c.484G>A of 76 Ghanaians whose genotypes were previously determined. The subjects with genotypes of G/G at c.484G>A are shown in blue, G/A in red, A/A in green. Negative control is shown in light blue.

According to our previous results [13], as well as the results of Erythrogene [11], two other SNPs, c.55G>A (Ala19Thr, rs146199130) and c.61C>T (synonymous SNP, rs28362460), were located in the 59T-probe sequence (Figure 1a). c.55G>A was present at a much lower frequency in Europeans and Africans, and c.61C>T appeared to be specific to Africans and Americans. Because 55A or 61T was located at the 59G allele, ten haplotype combinations were expected, but only seven samples could be subjected to probe-based FMCA analysis because DNA samples with the other three haplotype combinations were not available (Table 2). Probe-based FMCA analysis could clearly separate the seven subjects using a VIC/HEX/Yellow 555 filter (Figure 2a). Tm values for each peak are shown in Table 2.

Next, for c.314C>T, subjects with genotypes of C/C (wild type), with Tm about 70.5 °C, T/C, with two Tm peaks of about 61 °C and 70.5 °C, and T/T with Tm about 61 °C for c.314C>T, were distinguished from each other using a Cy5 filter (data not shown). Finally, for c.484G>A, subjects with genotypes of G/G (wild type) with a Tm of about 57.5 °C, G/A with two Tm peaks of about 57.5 °C and 64.5 °C, and A/A with a Tm of about 64.5 °C for c.484G>A, were also clearly distinguished from each other using the FAM filter (data not shown).

### 3.2. Validation of Probe-Based FMCA for Genotyping of Three SNPs

We then analyzed 106 Ghanaians and 100 Caucasians, and the results of c.59T>G (also c.55G>A and c.61C>T) and c.314C>T were in perfect accordance with previous Sanger sequencing results [13]. Representative melting curve genotyping results for c.59T>C and c.484G>A in Ghanaians are shown in Figure 2b,d, and for 314C>T in Caucasians in Figure 2c.

We confirmed the repeatability of the present triplex probe-based FMCA method by two independent assays of 106 Ghanaians and 100 Caucasians.

### 3.3. Inferred Le Phenotypes from Combinations of Three SNPs Genotypes

Although we did not perform Le phenotyping in the present subjects, we inferred Le phenotypes from combinations of three SNPs genotypes. Thirty-five of 106 Ghanaians (33%) and 10 of 100 Caucasians (10%) were likely to have Le-negative phenotype (Table 3). However, as shown in Table 4, a limitation of the present assay is that there is a possibility of misclassification of about 1–2% subjects in the estimation of the Le phenotype, including alleles with SNPs that have not yet been characterized.

## 4. Discussion

Evidence is accumulating to suggest that *FUT3* polymorphisms or Le phenotypes are associated with a variety of pathologic conditions, including *Helicobacter pylori* infection, ischemic heart diseases, intestinal infections, inflammatory bowel diseases, ankylosing spondylitis, COVID-19 susceptibility, and autoimmune neutropenia [20,21,22,23,24,25]. Large-scale replication studies are needed to confirm these associations, and reliable *FUT3* genotyping methods are desirable for this purpose.

Several methods for detection of *FUT3* SNPs, such as PCR-RFLP, PCR using sequence-specific primers (PCR-SSP), an allele-specific oligonucleotide hybridization method, TaqMan assay, Sanger sequencing of PCR amplicons, and a multiplex SNaPshot assay, were reported [13,22,26,27,28]. In addition, we have recently developed three independent high resolution melting (HRM) analyses that detect SNPs, c.13G>A, c.59T>G, and c.202T>C, in *FUT3* and probe-based FMCA genotyping at positions of c.508G>A and c.1067T>A using a unique fluorescence probe (Eprobe) [14,19,29]. Compared to other methods, HRM, probe-based FMCA, and TaqMan assay do not require post-PCR processing. The TaqMan assay is an established and frequently used method for SNP detection. However, it requires two dual-labeled probes to detect one SNP, whereas the probe-based FMCA requires only one dual-labeled probe to detect one SNP [15]. Accordingly, the general TaqMan assay requires multiple assays to detect SNPs at multiple sites, whereas probe-based FMCA allows multiplex assay that can detect SNPs at multiple sites in a single assay by employing one site-specific fluorescence probe. Therefore, probe-based FMCA has the potential to reduce assay cost and time compared to TaqMan assay.

HRM is an effective method for detecting heterozygotes of rare variants [30]. However, it does not seem to be suitable for detecting individual homozygotes for SNPs that are present at relatively high frequencies since the difference in their respective Tm values is often very small (usually less than 1 °C). On the other hand, probe-based FMCA is suitable for detection of the homozygotes for each of the relatively high-frequency SNP because of the large difference in Tm values (usually 4–10 °C) between homozygotes for each of them [16]. Eprobe-based FMCA is an excellent method to detect SNPs [29], but its disadvantage compared to the dual-labeled probe-based FMCA is that the multiplex assay is limited because, at present, only three fluorochromes (oxazole yellow, thiazole orange, and thiazole pink) can be used, and the cost of probe synthesis is high.

The presence of SNPs other than the target SNP in the probe sequences would affect the melting curve profile obtained by probe-based FMCA analysis. Therefore, we searched the Erythrogene database to see if the three probes contained other SNPs and found that only the probe for c.59T>G detection contained two SNPs (c.55G>A and c.61C>T). The c.55G>A was found in European and African populations with quite low frequency, and c.61C>T was found in Africans and Americans (4.24% in Africans, 1.30% in Americans, and 1.30% in global populations); 61T was likely to be in complete linkage disequilibrium with both 59G and 508A. Although the frequency of 55A was quite low, this SNP appeared to be linked with the 59G allele and with neither 508A nor 1067T in Europeans [13]. Probe-based FMCA is likely to be able to detect other SNPs even if they are nearby, while HRM seems to be difficult when other SNPs are nearby. Thus, for large-scale association studies of *FUT3*, examination probe-based FMCA is more suitable than HRM analysis. In fact, accurate genotyping of c.59T>G was difficult with HRM analysis, especially for African samples containing c.61C>T, in addition to c.59T>G [14]. However, in this probe-based FMCA, the allele containing c.61C>T (55G-59G-61T) was clearly distinguished from the other alleles (55G-59T-61C, 55G-59G-61C, and 55A-59G-61C).

Recently, we have developed a probe-based FMCA to identify the three major *FUT2* SNPs involved in Se enzyme inactivation [31]. Simultaneous implementation of two probe-based FMCA methods for detection of *FUT2* and *FUT3* SNPs would allow for more accurate estimation of Le phenotypes.

In conclusion, the present probe-based FMCA for c.59T>G, c.314C>T, and c.484G>A is valid and feasible for inference of Le phenotypes and for association studies of *FUT3* in populations around the world.

## Figures and Tables

**Table 1:** Primers and probes for detection of SNPs in *FUT3*.

Primer Sequences	Position of *FUT3*	Differences with *FUT5*	Differences with *FUT6*	Amplicon Length
Detection of c.59T>G				
forward primer: 5′-CCTCTCTCCTCTCTTCCCAGA-3′	−32 to −12 bp	1	2	179 bp
reverse primer: 5′-ACTGGGAGCCCTAGGGGAT-3′	129 to 147 bp	6	10	
59T-probe: 5′-HEX-TCTGGCCGCACTGCTATTTCAGC-BHQ1-3′	48 to 70 bp			
Detection of c.314C>T				
forward primer: 5′-TGTGGCTCTGTCCCGCTGT-3′	225 to 243 bp	3	5	140 bp
reverse primer: 5′-GGAGGCGTGACTTAGGGTTGG-3′	344 to 364 bp	8	8	
314C-probe: 5′-Cy5-ACAGGCAGACACGGTCATCGT-BHQ2-3′	303 to 323 bp			
Detection of c.484G>A				
forward primer: 5′-AGCCACCCCCTAACTGCCA-3′	413 to 431bp	6	9	158 bp
reverse primer: 5′-GGTCTTGGCCGAGAGGTTGA-3′	551 to 570 bp	0	0	
484A-probe: 5′-FAM-CAGCAACTCCGACATCTTCA-BHQ1-3′	480 to 499 bp			

The position of SNPs is shown by bold letters. “Differences with *FUT5* (or *FUT6)*” indicate the number of base differences in each of primer with corresponding *FUT5* and *FUT6* sequences.

**Table 2:** Haplotype combinations of 55-59-61 and observed Tm values of probe-based FMCA analysis for 59T>G.

	55-59-61/55-59-61	Tm (°C)
Subject 1	G-T-C/G-T-C	68.0
Subject 2	G-T-C/G-G-C	68.0/61.5
Subject 3	G-G-C/G-G-C	61.5
Subject 4	G-T-C/G-G-T	68.5/53.5
Subject 5	G-G-C/G-G-T	61.5/53.5
Subject 6	G-G-T/G-G-T	53.5
Subject 7	G-T-C/A-G-C	68.0/56.5
DNA not available	G-G-C/A-G-C	-
DNA not available	A-G-C/A-G-C	-
DNA not available	G-G-T/A-G-C	-

**Table 3:** Inferred Lewis genotypes and phenotypes from three SNPs.

c.59T>G	c.314C>T	c.484G>A	Lewis Genotype	Lewis Phenotype	Number of Ghanaians	Number of Caucasians
T/T	C/C	G/G	*Le/Le*	positive	23	51
T/T	C/C	G/A	*Le/le*	positive	23	0
T/G	C/C	G/A	*le/le*	negative	9	0
T/T	C/T	G/A	*le/le*	negative	7	0
T/T	C/C	A/A	*le/le*	negative	2	0
T/G	C/C	G/G	*Le/le*	positive	17	8
T/G	C/T	G/G	*le/le*	negative	3	7
G/G	C/C	G/G	*le/le*	negative	14	1
T/T	C/T	G/G	*Le/le*	positive	8	31
T/T	T/T	G/G	*le/le*	negative	0	2

59G contained 55A-59G-61C and 55G-59G-61T.

**Table 4:** *FUT3* alleles whose Lewis phenotype may be misjudged by the present FMCA method.

SNPs	Inferred	Actual	Frequency (%)					
			Africa	America	E. Asia	Europe	S. Asia	Global
59T-508A	*Le*	*le*	0.23	0	0.20	0	0.20	0.14
59T-1067A	*Le*	*le*	0.08	0	0.60	0.10	0.30	0.22
202C-314C	*Le*	*le*	0.38	0	0	0	0.40	0.18
59G-484A-1067A	*le*	*le*	0	0	0.10	0	0	0.02
Uncharacterized	*Le*	?	1.60	0.99	0.60	1.10	1.32	1.16

59T-508A: 59T alleles with 508A; 59T-1067A: 59T alleles with 1067A (including 59T-202C-314C-1067A with frequency of 0.10% in S. Asia); 202C-314C: 314C alleles with 202C (including 59T-202C-314C-1067A with frequency of 0.10% in S. Asia); 314T allele with 202T was not observed in the database; 59G-484A-1067A: This allele itself is le, but when subjected to test for *Le/le^59,484,1067^* was erroneously determined to be *le^59,1067^/le^484^* plus other SNPs; Uncharacterized: alleles with only uncharacterized SNPs; Any allele may contain alleles with or without other additional SNPs. E. Asia: East Asia, S. Asia: South Asia. Allele frequencies were obtained from Erythrogene [11]; "?" means undetermined (may contain both both Le and le).

## Data Availability

The data presented in this study are available on request from the corresponding author here.
